# Correlations between psychological tests and physiological responses during fear conditioning and renewal

**DOI:** 10.1186/2045-5380-2-16

**Published:** 2012-09-17

**Authors:** Karen G Martínez, Melissa Castro-Couch, José A Franco-Chaves, Brenda Ojeda-Arce, Gustavo Segura, Mohammed R Milad, Gregory J Quirk

**Affiliations:** 1 Department of Psychiatry, University of Puerto Rico School of Medicine, P.O. Box 365067, San Juan, PR, 00936, USA; 2 Department of Anatomy & Neurobiology, University of Puerto Rico School of Medicine, San Juan, PR, USA; 3 Clinical Psychology Program, Ponce School of Medicine, Ponce, PR, USA; 4 Clinical Psychology Program, Carlos Albizu University, San Juan, PR, USA; 5 Department of Psychiatry, Massachusetts General Hospital and Harvard Medical School, Charlestown, MA, USA

**Keywords:** Fear, Conditioning, Extinction, Anxiety, Skin conductance, Personality traits

## Abstract

**Background:**

Anxiety disorders are characterized by specific emotions, thoughts and physiological responses. Little is known, however, about the relationship between psychological/personality indices of anxiety responses to fear stimuli.

**Methods:**

We studied this relationship in healthy subjects by comparing scores on psychological and personality questionnaires with results of an experimental fear conditioning paradigm using a visual conditioned stimulus (CS). We measured skin conductance response (SCR) during habituation, conditioning, and extinction; subsequently testing for recall and renewal of fear 24 hours later.

**Results:**

We found that multiple regression models explained 45% of the variance during conditioning to the CS+, and 24% of the variance during renewal of fear to the CS+. Factors that explained conditioning included lower levels of conscientiousness, increased baseline reactivity (SCL), and response to the shock (UCR). Low levels of extraversion correlated with greater renewal. No model could be found to explain extinction learning or extinction recall to the CS+.

**Conclusions:**

The lack of correlation of fear extinction with personality and neuropsychological indices suggests that extinction may be less determined by trait variables and cognitive state, and may depend more on the subject’s current emotional state. The negative correlation between fear renewal and extraversion suggests that this personality characteristic may protect against post-treatment relapse of symptoms of anxiety disorders.

## Background

There is increasing evidence that people with anxiety disorders show exaggerated fear responses during experimental fear conditioning, in which a visual cue is paired with a mild shock to elicit increases in startle or skin conductance [1-5]. For example, subjects with post-traumatic stress disorder (PTSD) have shown increased fear conditioning [6], as well as deficient extinction of conditioned fear responses [7], and reduced recall of extinction memories [4]. One way to further understand the clinical relevance of conditioned fear responses is to determine their relation to anxiety phenotypes and personality traits. Anxious thoughts and behaviors can be assessed with questionnaires such as the Beck Anxiety Inventory (BAI) and the State-Trait Anxiety Inventory (STAI) [8]. Anxiety disorders have also been associated with personality characteristics such as neuroticism and extraversion [9], and decreased performance in cognitive processes such as conflict resolution and attention to threat [8].

Recent studies have begun to examine the relationship between some of these anxiety phenotypes and fear responses. Healthy subjects’ level of conditioning showed a positive correlation with trait anxiety, surveys of worry/avoidance, low extraversion, and high neuroticism [10-12]. Extinction has shown the opposite relationship with extraversion and neuroticism [12]. In addition, an anxious state is associated with decreased SCR response during fear conditioning and, conversely, elevated SCR response during extinction [13]. These studies focused exclusively on the response to the conditioned stimulus, and did not take into account responses to the unconditioned stimulus or differential learning (CS + minus CS-). Moreover, no prior study has attempted to explain the renewal of fear after extinction, which occurs with changes in context; an emerging model of clinical relapse [14].

To address these issues, we administered a battery of tests to healthy volunteers prior to fear conditioning, extinction, and renewal. To measure thoughts and behaviors associated with anxiety, we used the BAI and STAI. Personality characteristics were assessed with the NEO Five Factor Inventory (NEO-FFI). For cognitive processes, we used two Stroop-type conflict detection tests: 1) The Multi-Source Interference Task (MSIT), which activates conditioning-related areas of anterior cingulate cortex [15], and 2) the emotional Stroop (EST), which activates extinction-related areas of the cingulate and perigenual cortices [16]. We hypothesized that indices of anxiety (high STAI and BAI scores, high neuroticism, and low extraversion) would correlate positively with conditioning measures (i.e., conditioning, renewal), and negatively with extinction. The Stroop conflict tests, which reflect prefrontal engagement, might correlate with conditioning and/or extinction.

## Methods

### Participants

Healthy Puerto Rican subjects were recruited from the local community via advertisements. Exclusion criteria included a history of neurological conditions, current psychoactive medications, or Axis I diagnosis within the past 6 months. A Structured Clinical Interview for DMS-IV (SCID-I-RV) was used to confirm the absence of an Axis-I diagnosis. Subjects who failed to condition (2 or more trials with SCR > 0.05 μS, n = 2) or were outliers in their SCR values (greater than 2 standard deviations from the mean, n = 1) were also excluded. The final sample consisted of 46 subjects (30 females and 16 males) ranging in age from 21–57 (Mean age females = 28.5 ± 9.6; Mean age males = 25.4 ± 3.5 years). Written informed consent was obtained from all participants in accordance with the requirements of the Institutional Review Board at the University of Puerto Rico, School of Medicine and methods were approved as protocol A5280110.

### Neuropsychological tests

**Figure 1:**
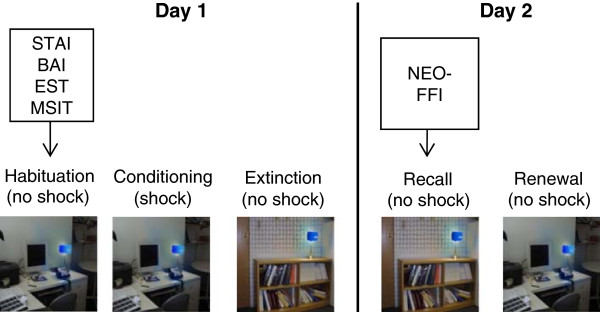
**Schematic of experimental protocol.** On each day, subjects performed psychological tests, and then underwent fear conditioning and extinction. Pictures of an office and a library represented conditioning and extinction contexts respectively, whereas the blue (or red) light represented the CS that was paired with the shock and later extinguished. STAI = State-Trait Anxiety Inventory, BAI = Beck Anxiety Inventory, EST = Emotional Stroop Task, MSIT = Multi-Source Interference Task, NEO-FFI = NEO Five Factor Personality Inventory.

All tests were validated for use with Spanish speaking subjects. Testing was performed over two days as outlined in Figure 1.

a. Anxiety symptoms scales

We used the BAI and STAI to measure anxiety symptoms. The BAI Spanish version used in our study has similar psychometric properties to the original English version [17,18] and the version of the STAI used for our study has been validated for Puerto Ricans [19].

b. NEO Five Factor Inventory (NEO-FFI)

The NEO-FFI is a 60-item self-report measure of personality traits across five dimensions: neuroticism, extraversion, agreeableness, conscientiousness and openness to experience [20]. The Spanish version of the NEO-FFI has been previously validated for Puerto Ricans [21]. Raw scores for each personality dimension were calculated and subsequently transformed into T-scores (M = 50, SD = 10) to adjust for sex differences.

c. Emotional Stroop Task (EST)

We designed a Spanish version of the EST using SuperLab software (Cedrus, Phoenix, Arizona). Participants were instructed to identify the ink color of a series of computer generated words as quickly as possible. Two types of words were used: threat-related and neutral words. The average reaction time (RT) was recorded by a key punch (ms), and a RT difference between threat and neutral words was calculated (RT threat minus RT neutral).

d. Multi-Source Interference Task (MSIT)

The MSIT is a numerical-based Stroop task measuring cognitive interference, designed to specifically activate the dorsal anterior cingulate cortex [15]. Subjects were presented with a set of three numbers (0, 1, 2, or 3), and were instructed to quickly select the one number that differed from the other two. In congruent trials, the position of the target number always matched the position of the key (i.e., 100, 020, 003). During interference trials, the position of the target never matched the position of the key (i.e., 010, 002, 300). Mean response latencies (in msec) for both congruent and incongruent trials were subtracted to yield a difference score (incongruent – congruent).

### Fear conditioning and extinction

We used a fear-learning paradigm previously described [22], in which subjects were tested over two consecutive days. On day 1, subjects completed habituation, conditioning, and extinction. The habituation phase consisted of 8 trials in which the conditioned stimuli (red or blue desk lights) were presented in two separate contextual (library and office), without any shock. The contextual stimulus appeared 6 sec prior to the onset of the desk light, which lasted for 12 sec, after which the screen went blank. The average inter-trial interval was 16 sec (range 12–21 sec). Habituation was immediately followed by conditioning, where one of the desk lights (e.g., red CS+) was paired with a mild shock to the second and third fingers of the dominant hand. Conditioning occurred within a specific context (e.g., office) (see Figure 1). The alternative desk light (e.g., blue CS-) was presented without any shock. The color of the stimulus for the CS + (blue vs. red light), as well as the conditioning context (library vs. office), were counter balanced across subjects. The shock lasted 0.5 sec and started immediately after CS + offset. The electric current was generated by a Coulbourn transcutaneous aversive finger stimulator (E13-22) powered by a 9-V dry cell battery. The intensity of the current was pre-set by each participant to be “highly annoying, but not painful”. The electrodes remained attached to the subject’s fingers during all phases of the experiment, and subjects were instructed prior to each phase that they “may or may not receive a shock”. Subjects were given 10 trials of conditioning (5 CS + and 5 CS-). After a few minutes, they received 20 trials of extinction (10 CS + and 10 CS-), in which the CS + and CS- were presented without shocks, in the alternate context (e.g., library).

On Day 2, subjects were brought back to the lab in order to test their retention of the extinction memory. Subjects were shown the context stimulus alone, followed 6 sec later by the CS + without any shock. Extinction memory was tested in two phases: recall and renewal, each consisting of 10 trials (5 CS + and 5 CS-). During the recall phase, the CS + was presented in the extinction context (recall of safety), whereas during the renewal phase, the CS + was presented in the conditioning context (recall of danger). The order of testing (recall vs. renewal) was counterbalanced across subjects.

### Physiological measures

The baseline skin conductance level (SCL) consisted of the average skin conductance during the 5 seconds prior to the first habituation trial. The skin conductance response (SCR) to each stimulus (CS + and CS-) was assessed as previously described [6,22,23]. The average skin conductance during the 6 sec context presentation was subtracted from the average skin conductance during the 12 sec CS + context presentation. We applied a minimum conditioning criteria that required subjects to show an SCR to the CS + greater than 0.05 μS in 2 or more conditioning trials [22,24].

### Statistical analyses

For each phase, the average SCR to the CS + and CS- were calculated as follows; 1) Habituation: average SCR of the first two trials habituation; 2) Conditioning: maximum SCR during conditioning; 3) Extinction: average SCR of trials 1–2 of extinction minus the average SCR of trials 9–10 of extinction; 4) Percent recall: average SCR in trials 1–2 of recall, divided by the peak SCR during conditioning; 5) Percent renewal: average SCR in trials 1–2 of renewal, divided by the peak SCR during conditioning [22]. Differential responses were calculated in the same way, except that the CS- was subtracted from the CS +.

ANOVAs with repeated measures were used to evaluate differences in SCRs to the CS + and CS-. Two-factor ANOVA’s (stimulus vs. trial) were performed for each phase of the experiment, to test for main effects and interactions. The stimulus main effect and stimulus X trial interaction effect were tested using the multivariate criterion of Wilk’s lambda (Λ). Paired-samples *t* tests were conducted to follow up the significant interactions. We controlled for familywise error rate across these tests by using Holm’s sequential Bonferroni approach (SPSS, version 19.0)

The relationship between psychological tests and physiological measures for each phase was evaluated using simple linear regressions, followed by stepwise multiple linear regression analysis using combinations of tests (SPSS, version 16.0). Simple linear regressions yielded an R^2^ representing the amount of variance explained by the test variable, and a beta coefficient (β) representing the correlation coefficient (r) between that test variable and the SCR. We performed a forward stepwise procedure in which variables were added to the model in two steps [25]. The first step included the psychological test variables: BAI, STAI, NEO personality factors, and the neuropsychological measures (MSIT, EST). The second step added additional variables that could have an effect on physiological responses such as sex, age, shock level, baseline SCL, and unconditioned responses to shock. This yielded a unique set of predictor variables for each phase. Multicollinearity between the predictor variables was evaluated by calculating the tolerance criteria [25]. β coefficients were determined for each factor in the model, and R^2^ values were adjusted for small sample size [25].

## Results

### Psychological test results

Our sample of healthy subjects scored within the normal range on all psychological and neuropsychological tests. Anxiety questionnaires showed no evidence of clinically relevant anxiety symptoms (mean BAI score = 3.5, mean state anxiety = 27.22%ile, mean trait anxiety = 30.52%ile) , and personality scores (mean neuroticism T score = 47, mean extraversion T score = 56 and mean conscientiousness T score = 47) were within normal limits [20]. Performance on the MSIT (mean reaction time = 340 msec) was similar to previous studies with healthy subjects [15]. The results of the EST showed no interference effect, on average, as expected from previous studies [26].

### Fear conditioning

#### Day 1: Habituation, conditioning and extinction

**Figure 2:**
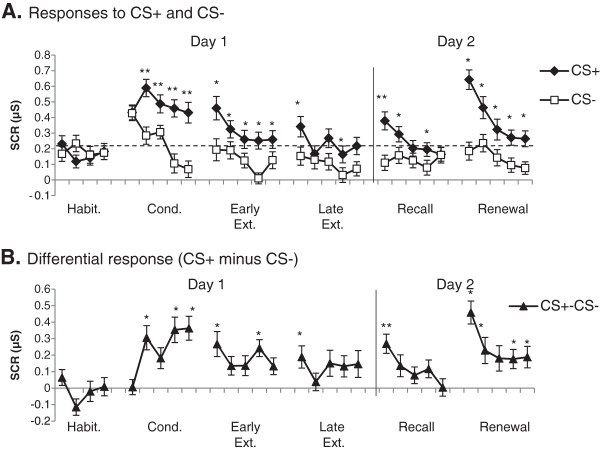
**Skin conductance responses (SCR) across all experimental phases. A**. Responses to CS + and CS- (single trials shown). On day 2, the order of recall and renewal was counterbalanced between subjects to correct for order effects. **B**. Differential SCR (CS + minus CS-). Differential learning in all phases was compared to last habituation trial. Habit. = Habituation, Cond. = Conditioning, Ext. = Extinction, μS = microsiemens, *p < 0.05; **p < 0.01.

During habituation, there were no differences in SCR to the CS + (stimuli paired with shock) and the CS- (stimuli never paired with shock) [Λ = .99, F_(1,45)_ = 0.48, p = 0.49]. During conditioning, responses to the CS + were significantly higher than those to the CS- (see Figure 2A). ANOVA during the conditioning phase revealed significant main effects for stimulus [Λ = .50, F_(1,45)_ = 44.24, p < 0.001] and trials [Λ = .77, F_(4, 42)_ = 3.20 , p = 0.022], as well as a significant stimulus X trial interaction [Λ = .62, F_(4,42)_ = 6.34, p < 0.001]. Post hoc comparisons revealed that CS + responses were significantly larger than CS- responses during trials 2–5 of conditioning (all p < 0.01), demonstrating successful acquisition of conditioned learning.

During the ten trials of extinction, responses to the CS + declined significantly. In extinction (trials 1–10), repeated measures ANOVA revealed significant main effects of stimulus [Λ = .64, F_(1,45)_ = 25.91, p < 0.001] and trials [Λ = .67, F_(9,37)_ = 2.01, p = 0.065] and a non-significant stimulus X trial interaction [Λ = .70, F_(9,37)_ = 1.73, p = 0.115]. Post hoc comparisons revealed that responses to the CS + remained significantly larger than to CS- during all early extinction trials (all p < 0.02). During late extinction, significant differences were observed for trials 6 and 9 only (both p < 0.05).

#### Day 2: Recall of extinction and renewal

The following day, subjects were returned to the laboratory to test for recall and renewal, in counterbalanced order. During the recall phase, in which the CS + was presented in the extinction context, ANOVA revealed a significant main effect of stimulus [Λ = .73, F_(1,45)_ = 16.67, p < 0.001], but not trials [Λ = .682, F _(4,42)_ = 2.24, p = 0.081], and a significant interaction [Λ = .71, F_(4,42)_ = 4.27, p = 0.05]. Post-hoc analysis showed that the response to the CS + was significantly higher than to the CS- in trials 1 (p < 0.001), 2 (p = 0.047) and 4 (p = 0.036). Responses to the CS + returned to habituation baseline by the fifth trial.

During the renewal phase, in which the CS + was presented in the conditioning context, the responses to the CS + dramatically increased. ANOVA revealed significant main effects of stimulus [Λ = .58, F_(1,45)_ = 31.47, p < 0.001] and trials [Λ = .59, F_(4,42)_ = 7.32, p < 0.001], with a significant stimulus X trial interaction [Λ = .72, F_(4,42)_ = 4.01, p = 0.008]. Post hoc comparisons revealed that responses to the CS + were significantly larger than to the CS- on all renewal trials (all p < 0.05) demonstrating prolonged effects of altering the context.

### Differential response

The differential response (CS + minus CS-) reflects the degree of associative learning to the CS + (Figure 2B). Compared with the last trial of habituation, pairwise comparisons revealed significant differential responding during trials 2, 4, and 5 of conditioning (all p < 0.01), trials 1, 4, and 6 of extinction (all p < 0.05), the first trial of recall (p = 0.005), and trials 1, 2, 4, and 5 of renewal (all p < 0.05).

### Regression analysis

#### Simple linear regression

We first used simple linear regressions to evaluate the degree to which individual tests could account for variance in SCL or SCR in the different phases, calculating a regression coefficient (R^2^). Most psychological measures were not significantly correlated with SCL or SCR responses in any experimental phase (see Table 1). When significant correlations did occur, they accounted for only 8 to 15% of SCR variance. The variance of SCL (15%) was explained by extraversion (ß = −0.41, p = 0.005). For conditioning memory, conscientiousness could explain 7% of the variance (ß = −0.30, p = 0.043), whereas for extinction learning, the EST could explain 7% of the variance (ß = 0.30, p = 0.036). Percent renewal showed similar weak correlations with extraversion (ß = −0.413, p = 0.004; 17%). No test could account for significant variance in recall or habituation phases.

**Table 1:** Results of simple regressions for CS + responses

** Tests**	**SCL**	**Cond.**	**Extinction**	**Recall**	**Renewal**
	**R**^**2**^	**R**^**2**^	**R**^**2**^	**R**^**2**^	**R**^**2**^
Trait Anxiety	−0.022	0.009	0.022	−0.008	−0.023
State Anxiety	0.056	0.013	0.004	−0.005	−0.018
Beck Anxiety	−0.020	0.003	0.007	0.001	0.037
Neuroticism	0.021	0.000	0.041	0.000	0.000
Extraversion	0.145**	0.001	0.016	0.063	0.170**
	β = −0.405				
					β = −0.413
Conscientiousness	−0.021	0.071*	0.063	0.019	0.019
		β = −0.304			
EST Reaction Time	−0.023	0.001	0.072*	0.035	0.008
			β = 0.304		
MSIT Reaction Time	−0.010	0.028	0.009	0.037	0.014

Shown are values for *R*^2^ (the amount of SCR variance accounted for each test). For significant correlations with SCR, β is shown. SCL = baseline skin conductance level, Cond. = Conditioning, EST = Emotional Stroop Task, MSIT = Multi-Source Interference Task. *p < 0.05, **p < 0.001.

#### Multiple regression

**Figure 3:**
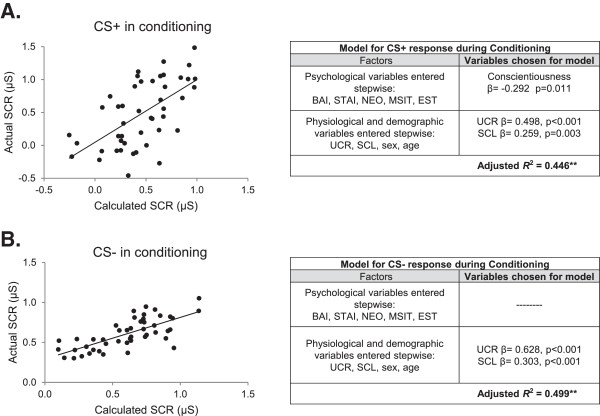
**Multiple regression models for conditioning.A**. Model for CS+. **B**. Model for CS-. Plots compare the correlations between the actual CS responses and those calculated by the multiple regression model. μS = microsiemens, **p < 0.01.

We next conducted multiple regression analyses, using a stepwise approach. The possible predictor variables were added to the model in two steps [25]. The first step included the psychological, personality, and neuropsychological test variables. A second step added additional variables that could have an effect on physiological responses such as sex, age, shock level, baseline SCL, and unconditioned responses to shock. This process yielded a unique set of predictor variables for each phase. For the conditioning phase (peak conditioning), a significant model was found which accounted for 45% of the SCR variance across subjects (adjusted R^2^ = 0.446, p < 0.001) (see Figure 3A). Significant variables in the model included one personality trait (conscientiousness) and two physiological variables (i.e., UCR, SCL). Lower levels of conditioning were correlated with higher conscientiousness scores and lower UCR and SCL levels. Multicollinearity analysis showed that correlations between the predictor values was not a factor affecting the conditioning model (tolerance scores = 0.931-0.988). For the CS- responses, a significant model was found for peak conditioning consisting only of physiological variables (i.e., UCR, SCL), explaining 50% of the response (Adjusted R^2^ = 0.499; p < 0.001, see Figure 3B).

**Figure 4:**
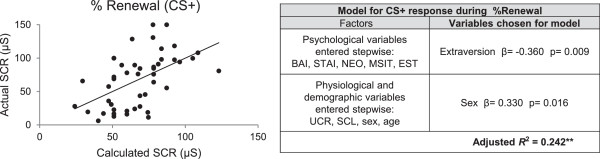
**Multiple regression model for CS + during renewal.** Plot shows correlations between the actual SCR responses and those calculated by the multiple regression model for % fear renewal**.** μS = microsiemens, **p < 0.001.

No models were found that correlated with extinction learning or extinction recall. For the renewal phase, however, a model was found to account for 24% of intersubject variance in CS + response (Adjusted R^2^ = 0.242, p < 0.001) (see Figure 4). The model consisted of a negative correlation with extraversion and a positive correlation with sex (i.e., being male associated with higher renewal). This model also showed appropriate tolerance criteria (0.945-0.969) minimizing the risk of multicollinearity. There was also a significant (but weaker) model for CS- responses in renewal, using only physiological variables UCR and SCL (Adjusted R^2^ = 0.163; p = 0.008).

## Discussion

We have assessed the relationship of several psychological tests related to anxiety phenotypes with physiological indices, both at baseline and during various phases of experimental fear conditioning. While we were unable to find correlations with extinction learning or extinction recall, we were able to explain 24 to 45% of variance in SCR responses during conditioning and renewal, both indicators of the strength of the CS-US association.

The predictive power of our model for conditioning (45%) is consistent with findings from Otto and colleagues, who were able to account for 28% of CS + variance in conditioning, also using multiple regression [10]. This is interesting given that the two models employed different measures. While our model included only conscientiousness and physiological variables associated with reactivity, Otto et al. (2007) found a predictive model that included the Penn State Worry Questionnaire (PSWQ), the Fear Questionnaire (FQ), and the Anxiety Sensitivity Index (ASI). Conscientiousness can be related to PSWQ scores, in that it assesses thoughtfulness and carefulness [20] , and was previously reported to be correlated with conditioning [9]. Thus, together with Otto et al. (2007), we suggest that approximately 28-45% of SCR variance in conditioning may be due to individual characteristics that can be revealed with self-report scales and measures of physiological reactivity. The greater variance explained in our sample was probably due to our taking into account response to the shock and baseline skin conductance.

In contrast to conditioning, we were unable to find significant correlations with extinction learning or extinction recall, using either linear or multiple regression. This differs from Rauch et al. (2005) who reported that recall of extinction was correlated with extraversion (r = 0.77) and neuroticism (r = −0.61) [12]. The experimental protocols used at the two sites were designed to be identical. Factors that might account for the discrepancy include the larger number of subjects in the current study (46 vs. 14 in Rauch et al.), or an ethnic factor affecting the relationship between personality and extinction (Puerto Rican vs. a largely Caucasian sample in the Boston area). A lack of correlation of extinction with psychological and neuropsychological indices suggests that extinction may be less determined by trait variables, and may depend more on the subject’s current emotional state. In support of this idea, Milad et al. (2008) showed that extinction retention deficits in PTSD patients were acquired rather than familial, based on a twin design [27]. Additionally, Vriends et al. (2011) reported decreased extinction in healthy participants who first watched a film intended to induce an anxious emotional state [13]. Our state measure (STAI) showed no correlation with extinction, likely due to the low state anxiety levels of our healthy subjects in the absence of a provocation such as that used in Vriends et al. (2011).

Our most novel finding was the ability to successfully model contextual renewal. Renewal of extinguished fear with a contextual shift is thought to model relapse after treatment with exposure therapy [14]. It has been suggested that the ability to successfully inhibit fear responses when challenged by contextual shifts is the best predictor of clinical response [28]. Extraversion was negatively correlated with renewal, consistent with a protective effect. Individuals with high extraversion scores are more likely to seek stimulation, undertake activities with unknown consequences, and experience positive emotions [20,29]. Extraversion is also positively correlated with the volume of the medial oribitofrontal cortex [30], an area implicated in fear inhibition [31] and reward [32]. Thus, the presence of extraversion may reflect the engagement of structures that modulate fear responses under ambiguous conditions. In addition, sex was also a significant factor in the model (males showed higher renewal). This is an interesting finding given that anxiety disorders are more prevalent in females [8,33]. We are now conducting additional studies on the sex differences we observed in our sample.

We propose several follow-up steps to extend these findings and to address the limitations of our study. In order to state that personality characteristics can predict physiological responses, the predictive power of these models should be tested using predictive model analysis with new subjects. The applicability of these results to subjects with anxiety disorders should also be evaluated. It will be particularly interesting to determine the extent to which conditioning and renewal models are able to predict the severity of anxiety disorders, response to treatment, and/or post-treatment relapse, in longitudinal studies. Factors that could be predictive of renewal, such as low extraversion in our sample, may be used for identifying patients at risk for relapse, who might benefit from preventive measures such as delivering therapy in multiple contexts or providing reminders of the therapeutic context [34].

## Conclusion

Using models that included personality characteristics previously associated with the anxiety phenotype as well as physiological responses, we were able to explain up to 45% of variance in experimental fear conditioning and renewal. Understanding the relationships between personality characteristics and fear learning could help in the development of markers to identify people at risk for anxiety disorders and/or risk of relapse after treatment.

## Competing interests

The authors declare that they have no competing interests.

## Authors’ contributions

KGM and MC-C participated in the development of the methodology, implementation of research protocol, statistical analysis and preparation of the manuscript. JAF-C participated in the development of the methodology, implementation of research protocol and statistical analysis. BO-A and GS collected data. MRM and GJQ contributed in the development of the methodology, interpretation of results and preparation of the manuscript. All authors read and approved the final manuscript.
